# An Analysis of Volume, Length and Segmentation of Free Fibula Flap in Reconstruction of the Jaws: Investigation of Their Role on Flap Failure

**DOI:** 10.3390/reports6010004

**Published:** 2023-01-29

**Authors:** Mattia Di Bartolomeo, Irene Laura Lusetti, Massimo Pinelli, Sara Negrello, Arrigo Pellacani, Stefano Angelini, Luigi Chiarini, Riccardo Nocini, Giorgio De Santis, Alexandre Anesi

**Affiliations:** 1 Surgery, Dentistry, Maternity and Infant Department, Unit of Dentistry and Maxillo-Facial Surgery, University of Verona, P.le L.A. Scuro 10, 37134 Verona, Italy; 2 Plastic Surgery, University of Modena e Reggio, Policlinico di Modena, 41124 Modena, Italy; 3 Cranio-Maxillo-Facial Surgery Unit, University Hospital of Modena, 41124 Modena, Italy; 4 U.O.C. Hematology and Cellular Therapy, Ospedale Mazzoni, 63100 Ascoli Piceno, Italy; 5 Department of Medical and Surgical Sciences for Children & Adults, Cranio-Maxillo-Facial Surgery, University of Modena and Reggio Emilia, Largo del Pozzo 71, 41124 Modena, Italy; 6 Section of Ear Nose and Throat (ENT), Department of Surgical Sciences, Dentistry, Gynecology and Pediatrics, University of Verona, 37124 Verona, Italy

**Keywords:** fibula flap, free flap, mandible reconstruction, maxillary reconstruction, head and neck reconstruction, complications, free flap failure, segmentation, free fibula flap

## Abstract

Reconstruction of defects of the jaws is mainly performed via free fibula flap. An incidence of 2–21% of overall flap failure is still described. We investigated the roles of volume, length and number of fibula flap segments on flap survival using novel three-dimensional segmentation tools. We also analyzed the role of other possible risk factors. Seventy-one consecutive patients with a follow up of at least three months and who underwent free fibula flap reconstruction in a single center between 2002 and 2022 have been evaluated. A total of 166 fibula segments were analyzed. Malignancies were the main reason of resection (45.1%). In 69% of the cases a reconstruction of the mandible was performed. The flaps were mainly divided in two segments (39%) (range 1–4), with a mean length of 2.52 cm and a mean volume was 3.37 cm^3^. Total flap failure (TFF) occurred in 12 cases, (16.9%), while partial flap failure (PFF) appeared in 3 patients (4.2%). Volume, length and number of fibula flap segments did not seem to influence flap failure incidence in uni- and multivariate analysis. Reconstruction of the maxilla and use of a recipient vessel different from the facial artery seemed to significantly impact on flap failure. Smoking and previous surgeries showed a higher trend to flap failure, but they did not reach statistical significance. Prospective and multicentric analysis on a wider population should be assessed.

## 1. Introduction

Reconstruction of the jaws is a topic of ample and transversal interest. At the present moment, the reconstructive gold standard in those cases of extensive jaws osseous defect is represented by the free fibula flap [1]. Other microsurgical options include iliac crest and scapula flaps, each one with specific pros and cons [2]. The advantages of free fibula flap include:

- A quite standardized harvesting technique;
- A constant and regular vascular anatomy;
- The large amount of bone, that allows the reconstruction of large deficits;
- The possibility to perform many osteotomies in order to model the flap and to reach a more morphologic/aesthetical and functional result;
- The possibility to position endosseous dental implants, that will subsequently allow a prosthetic reconstruction;
- The possibility to harvest an osteo-myo-cutaneous flap, thus allowing to reconstruct also the soft tissues;
- The reduced morbidity of the donor site [1].

As in other free flaps, the main risk is represented by the vascular thrombosis, which leads to a flap failure. In fibula flaps, a partial flap failure (PFF) or a total flap failure (TFF) are described. In the literature, this complication ranges between 2% and 21% [3,4,5,6,7,8,9,10,11,12,13,14,15,16]. Causal factors leading to flap failure mainly remain speculative. Several risk factors are described [7,9,10,17,18,19,20], such as:

- Smoking habits;
- Alcohol consume (which can act both in a direct and/or indirect manner);
- Pro-thrombotic conditions;
- Diabetes mellitus;
- Previous therapies, such as radiotherapy and/or chemotherapy;
- Malnutrition;
- Body mass index (BMI);
- Male sex;
- Intraoperatory time.

Nonetheless, a clear role and how much these factors affect flap survival is not described. The segmentation of the fibula should be examined as well. In fact, the number and the length of bone segments could be involved in free flap survival. As clearly demonstrated by Fichter et al., a greater length and a lower number of osteotomies positively correlated with bone perfusion. The longer endosteal vascularization and the wider periosteal area were thought to be among the contributing factors [9,21]. The detachment of the periosteum is required in proximity to osteotomic lines when harvesting and segmenting the flap. The periosteal layer plays a key-role in vascularization of the segment and the more it is detached, the more there is the risk of segment necrosis, especially in smaller segments. Besides these bidimensional considerations, a three-dimensional evaluation with new technologies could be helpful as well, both in the planning of the resection and in the customization and segmentation of the flap [16,22,23,24,25,26]. In fact, the volume of the segment can be an influencing factor of the final result. A shorter segment will have a smaller periosteal area. Moreover, as we have seen before, multiple osteotomic lines can put at risk flap survival, which has to rely more on periosteal blood vascularization. Recently, reliable segmentation tools have been developed, allowing the volumetric measurement of each segment. To the best of our knowledge, the role of the volume of the segments has never been investigated before.

Given these considerations, we decided to evaluate the role of volume, length and number of segments in free fibula flap reconstruction of the jaws. Moreover, we also performed a univariate and multivariate analysis in order to study other possible influencing variables.

## 2. Materials and Methods

### 2.1. Study Design and Data Collected

In this retrospective analysis, we reviewed consecutive patients who underwent free fibula flap reconstruction of the jaws between 2002 and 2022. Surgical interventions were conducted in collaboration between the Department of Cranio-Maxillo-Facial Surgery and the Department of Plastic and Reconstructive Surgery of the University Hospital of Modena, Italy (Azienda Ospedaliero-Universitaria di Modena). Free fibula flap harvesting was performed according to our previously described technique, which is here briefly summarized [27]. The choice of the laterality of the leg was made preoperatively, according to:

- A computed tomography angiography, to study the anatomical variations of the peroneal artery.
- The planning of the resection.
- The possible need of a skin paddle.
- The evaluation regarding the localization of the pedicle.

The patient was put in a supine position and the selected knee was bended, thus allowing a simultaneous two-team approach. A classical lateral access was performed to harvest the flap, with or without tourniquet according to the surgeon’s preferences [28].

Demographic, clinical, surgical (preoperative, intraoperative and postoperative) and radiological data were collected. All the patients included had a minimum clinical follow up of six months.

For all patients the following data were recorded: smoke and alcohol consume habits, comorbidities (diabetes, hypertension, cardiovascular diseases, chronic pulmonary insufficiency, autoimmune diseases, osteoporosis, immunodepression, hypovitaminosis D, coagulopathy, hypocalcemia, hyperparathyroidism and kidney failure), ASA score, BMI, previous treatments (chemotherapy, radiotherapy and/or surgery). Intraoperative variables were collected as well: number of fibula flap segments used for reconstruction, recipient artery and veins, use of single or double vein anastomosis, operation time, simultaneous neck dissection and/or tracheotomy. When feasible, a post-operative orthopantomography (OPG) or computed tomography (CT) was performed in order to radiologically evaluate the reconstruction site. In those cases where a CT scan was available, we measured the length of every fibula flap segment using the free, open-source medical imaging viewing software Horos (Lesser General Public License (LGPL) 3.0). Regarding the volume, each fibula flap segment section was measured on every single CT scan and the final volume was then calculated. The software Horos (Lesser General Public License (LGPL) 3.0) has been used in this case as well.

Flap failure was divided in two groups. In the total flap failure (TFF) group all the patients that underwent a complete flap removal were included. On the contrary, partial flap failure (PFF) was defined when at least one fibula flap segment was salvaged.

### 2.2. Statistical Analyses

A descriptive analysis of all variables was performed including mean, median, standard deviation, range, minimum and maximum value for continuous variables, absolute and relative frequencies for categorical variables.

Univariate analysis for identifying risk factors was performed with a generalized linear model for binary outcomes with a logit link fit to model the relation between the above-mentioned variables and failure.

The joint effect of variables on flap failure was evaluated using the multivariate logistic model and a stepwise model selection was used to find the combination of explanatory variables that had the best relationship with failure.

A *p*-value of 0.05 was used as the criterion for statistical significance. The analysis was conducted using R Version 3.6.0 (www.r-project.org).

## 3. Results

From January 2002 to June 2022, a total of 71 cases of microsurgical reconstruction of the jaws with free fibula flap were performed. The patients ranged in age between 15 and 74 years, with a mean age of 47.6 years. The mean BMI was 24.6 kg/m^2^. Further patients’ features are described in Table 1.

**Figure 1:**
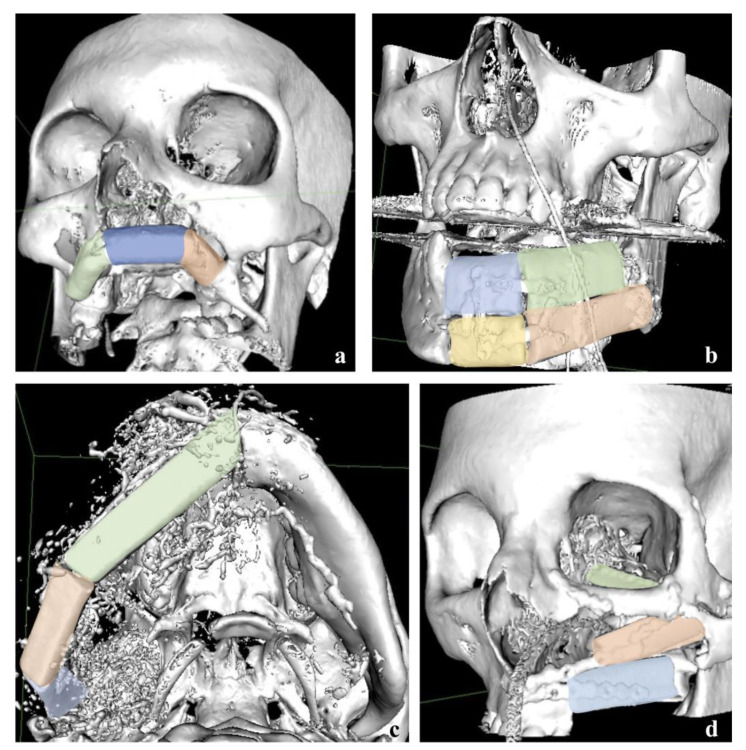
Examples of challenging reconstructive cases. (**a**) Three-pieces reconstruction of the whole maxilla due to excision of a chondrosarcoma in a young girl. (**b**) Double-barreled reconstruction of left body of the mandible following removal of an ameloblastoma. (**c**) Reconstruction of right hemimandible with free fibula flap in a three-pieces fashion. A widely extended arterio-venous malformation was removed after its embolization. Embolizing material artifacts are visible in the CT. (**d**) Midface reconstruction achieved by means of a free fibula flap in three segments after having removed a massive psammomatoid ossifying fibroma.

A large cohort of patients underwent surgical and/or medical treatment before fibula flap reconstruction: at least one surgical intervention in the head and neck district was performed before in 46.5% of the cases, while 22.5% and 17.0% of the patients underwent radiotherapy or chemotherapy prior to surgery, respectively. Table 1 shows that the main indication for surgery was represented by malignant and benign oncological disease (45.1% and 23.9%, respectively), followed by osteoradionecrosis (8.5%). Among malignancies, oral squamous cell carcinoma was the most frequent diagnosis and it required a prompt intervention even during COVID-19 pandemic, but rare conditions such as a primary intraosseous squamous cell carcinoma (PIOSCC) were treated as well [29,30]. Other indications for surgery were vascular malformations, gunshot, medication-related osteonecrosis of the jaws (MRONJ), atrophic jaws and osteomyelitis. A few examples of challenging reconstructive cases are illustrated in Figure 1, below.

In almost two-thirds of the cases the reconstructive intervention involved the mandible (66.2%), while in 31.0% the maxilla. In two patients a combined maxillo-mandibular reconstruction was performed. The mean operative time was 11.5 h, ranging from a minimum of 6.5 to a maximum of 18 h. In 25.4% of the patients a simultaneous mono- or bilateral neck dissection was performed, while a simultaneous tracheotomy was needed in 66.2% of the cases in order to protect the airways.

Regarding recipient vessels, the facial artery was the mainly used one (69.0%). In 11 cases, we were not able to track down the recipient artery. In the remaining cohort, the external carotid artery and the superior thyroid artery were mainly chosen, followed by the lingual artery and the submandibular artery. Regarding the recipient veins, unfortunately in 32.4% of the cases this aspect was not indicated in surgical reports. In other cases, a single-vein anastomosis was performed in 26 times (36.6%) and a double-vein anastomosis was made in 22 cases (31.0%).

**Figure 2:**
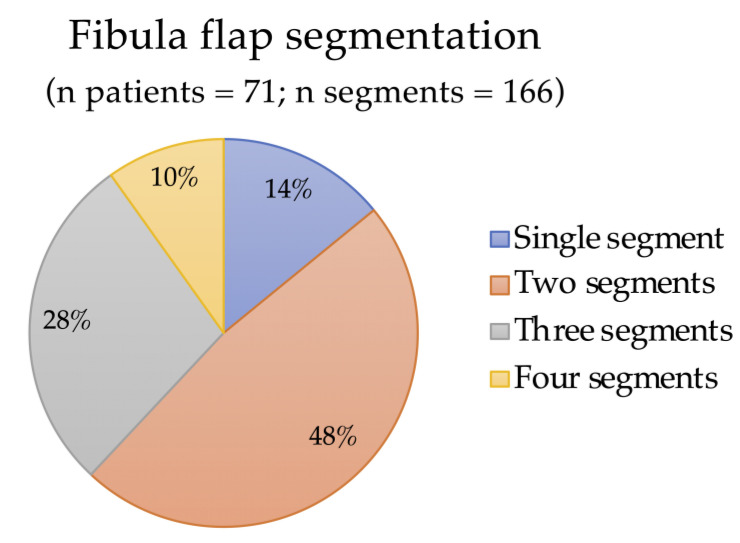
Fibula flap segments distribution.

Concerning the measurements of fibula flap segments, a total of 166 segments were used. The mean number per patient was 2.4, with a maximum number of 4 and a median number of 2 segments. The distribution of the segments is depicted in Figure 2. Overall, 63.3% (*n* = 45) of the patients underwent at least one CT scan in the post-operative period. Among them, the longest segment measured 9.4 cm and the mean length was 2.5 cm. The mean volume per segment was 3.3 cm^3^, with the biggest one measuring 18.8 cm^3^.

**Figure 3:**
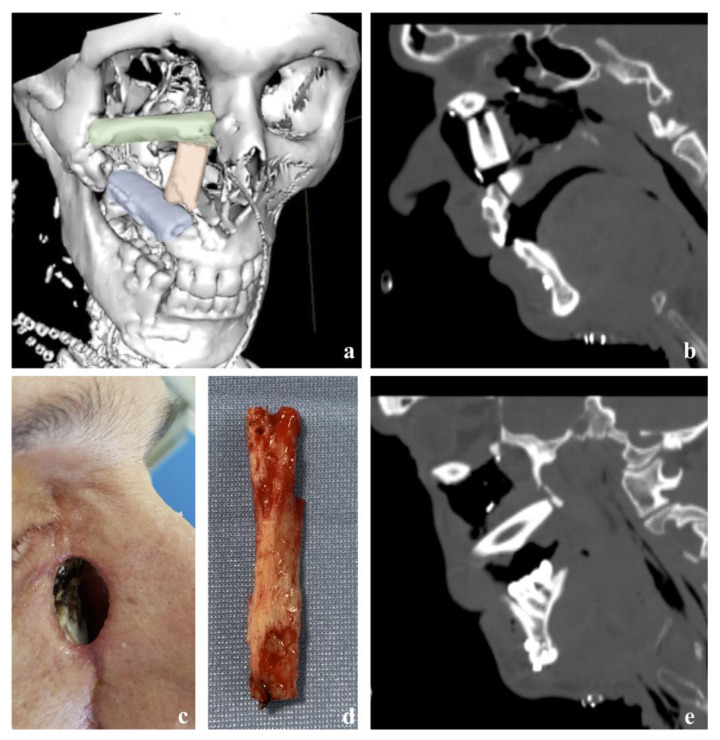
Partial flap failure of a free fibula flap used to reconstruct the left midface following the excision of a SCC originated from the maxillary sinus, with orbital invasion. (**a**) Three-dimensional view of the reconstruction. The pink segment, used to reconstruct the naso-maxillary buttress, was the one that subsequently had to be removed. (**b**) Post-operative CT showing the naso-maxillary segment. (**c**) Naso-cutaneous fistula determined by super-infection of the necrotic segment. (**d**) Naso-maxillary segment removed on post-operative day (POD) 23. (**e**) Post-operative CT showing the absence of the removed segment.

**Figure 4:**
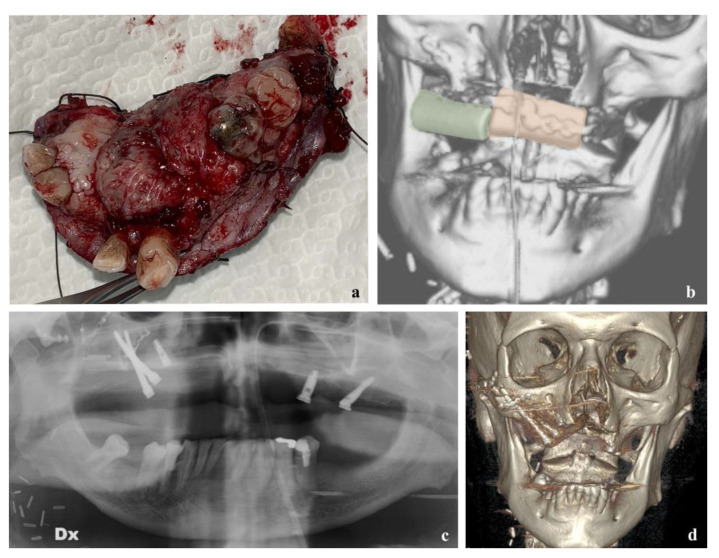
Total flap failure in a patient operated to remove an oral SCC of the maxilla. The patient was affected by epidermolysis bullosa, an autoimmune disease which might have co-played a role in flap failure. (**a**) Right hemi-maxillectomy, including the carcinoma. (**b**) Three-dimensional view of the reconstruction achieved by a two-pieces fibula flap. (**c**) Post-operative OPG showing absence of the necrotic segments. Total flap failure occurred on POD 13. The removal of the necrotic flap was followed by positioning of two right zygomatic implants, three traditional endosseous implants and a temporalis flap. (**d**) Three-dimensional post-operative CT showing the correct positioning of the implants and the osseous defect.

Focusing on post-operative results, a 4.2% (*n* = 3) incidence of partial flap failure (PFF) and a 16.9% (*n* = 12) incidence of total flap failure (TFF) were reported. In all cases of partial flap failure, the indication for surgery was due to widely extended malignancies of the maxilla. Among patients who experienced TFF, these data decreased to half of the cohort. Clinical examples of PFF and TFF are reported in Figure 3 and Figure 4.

Univariate analysis showed a significant relation between maxilla site and flap failure (*p* < 0.001) and between a non-facial artery used as a recipient vessel and flap failure (*p* = 0.043). No other statistically significant risk factors could be identified. Results of the univariate analysis are described in Table 2.

Multivariate analysis showed that smoking (*p* = 0.124), maxilla site (*p* = 0.067), a non-facial artery used as a recipient vessel (*p* = 0.099) and previous surgical interventions (*p* = 0.089) tended to a higher risk of flap failure, although these results were not statistically significant.

## 4. Discussion

As of today, revascularized free fibula flap represents the main option for major surgical reconstruction of the jaws. Nonetheless, in the literature the flap failure incidence ranges between 2% and 21%, rising great attention on those factors that can affect flap survival [3,4,5,6,7,8,9,10,11,12,13,14,15,16].

In our cohort, we have experienced a 4.2% (*n* = 3) PFF incidence and a 16.9% (*n* = 12) TFF incidence. There are, however, a few possible explanations. In almost half of the patients (45.1%; *n* = 32) the surgical intervention was required due to a widely extended malignancy and in 8.5% of the cases a free fibula flap was performed because of a major ORN. In accordance with the present results, previous studies have demonstrated that these conditions might increase the risk of flap failure [17,31]. In fact, in both cases a disruption of the normal anatomy occurs. Moreover, radiotherapy is followed by several modifications of the vessels in the recipient site: perivascular fibrosis, endothelial damage and intima thickening were described, thus leading to an alteration of the blood flow and ultimately to vascular thrombosis [32,33,34]. Concerning the disruption of the regular anatomy, in our analysis we noted that those cases when previous surgical interventions were performed tended to a higher risk of flap failure (*p* = 0.089), although without reaching statistical significance. Bouland also reported a higher flap failure rate in those patients that previously underwent neck dissection, as well as in previously irradiated patients [35]. Previous surgical interventions, especially on the neck, create perivascular fibrous adherences. The consequence is a more laborious preparation of arteries and veins, increasing the manipulation time of the vessels in order to adequately prepare the recipient site. Given these considerations, it is not surprising that the overall flap failure incidence in our populations trends towards the higher limit. At the same time, despite being a limited number of cases, further considerations on other factors that might affect flap failure can be raised.

Following the recent development of reliable, three-dimensional segmentation tools, we decided to focus our attention on the possible influence of the volumes of the segments on flap survival. Moreover, we have also decided to evaluate the eventual role of the number and the length of the segments. To the best of our knowledge, the role of the volume of the segments on fibula flap survival was never analyzed before. For length and volume measurements, we used the free, open-source medical imaging viewing software Horos (Lesser General Public License (LGPL) 3.0). Univariate and multivariate analysis were performed. In our paper the volume, length and number of osteotomized segment did not seem to influence flap failure. The reason should probably be found in the meticulous surgical technique applied and in the long surgical experience gained in this field. In fact, together with the team of prof. Hidalgo and prof. Cordeiro, our team was among the first to start the reconstruction of the jaws with free fibula flaps [27,36]. From the beginning of our experience, the surgical technique required a scrupulous maintenance of the periosteal layer adherent to the bone cortex, in order to keep the periosteal vascularization as intact as possible. The periostium is detached only close to the osteotomy line and it is limited to the minimum. Moreover, the research on bone healing mechanisms has allowed substantial ameliorations to surgical techniques [37]. With the recent development of piezosurgical instruments, we have progressively increased their use at the expense of rotary instruments. As we have demonstrated on animal models, piezosurgical osteotomes allow to reduce the bone gap, fasten bone healing and increase bone regeneration and remodeling [38]. Together with the care used to manipulate soft tissues (including the vascular pedicle), the attention to preserve hard tissues is crucial in a free fibula flap reconstructive intervention.

Another aspect that to be considered is that volumetric data were not available for all patients. In fact, less than 50% (*n* = 7) of patients who underwent flap failure was able to undergo a post-operative CT. Among all patients, 63.3% of the patients underwent a CT after the surgery, while 70.4% underwent an OPG. Overall, only 16.9% (*n* = 12) of the patients was not able to undergo a radiographic exam to evaluate the osteointegration of the flap and more than half of these patients experienced TFF. This issue was more common in immediate flap failure cases, due to the absence of time to perform the exam and due to the absence of an intraoperatory CT given its high costs. Sometimes, the instability of clinical conditions did not allow us to obtain a post-operative CT. Finally, it must be underlined that, according to the literature, at the beginning of the present case series, an OPG was considered a proper exam to immediately evaluate osteointegration. However, OPG is not considered reliable for linear measurements due to the distortions determined in the acquisition process [39]. For these reasons, we have recently implemented our protocol to program a post-surgical CT in the first PODs.

Focusing on other risk factors considered, it has to be noted that the choice of the recipient artery seemed to influence flap failure (*p* = 0.043) in our univariate analysis. In 11 cases (15.5%) it was not possible to detect these data on previous surgical reports, while in the remaining population (84.5%) a clear dichotomy between the choice or not of the facial artery was evident. In fact, the facial artery was used as the recipient one in 49 patients (69%). This finding is in discrepancy with the actual trend described in literature, as some recent authors have expressed their preference towards the use of superior thyroid artery as a recipient vessel [40,41]. We used it as a recipient artery as well, together with the external carotid, the lingual artery and the submandibular artery (total *n* = 11). In this population, we have noticed two different groups:

- In the first group, we had to choose an artery different from the facial one due to oncological radicality (*n* = 7) and hence the need for a facial artery ligation. Among them, in 5 times a partial or total flap failure occurred.
- In the second group, the choice was made after a thorough intraoperatory evaluation. The indications for surgery were ORN (*n* = 3) or the ablation of a wide arterio-venous malformation (*n* = 1). No flap failure occurred in this group.

Despite a very limited number of patients, this finding supports the role of tumoral extension as a possible risk factor for flap failure.

Intraoperatory time, age and BMI were also described as possible risk factors for flap failure [7,9,10,17,18,20]. Nonetheless, in our paper we did not find a correlation between them and PFF or TFF. On the contrary, smoking habits seemed to predispose to failure occurrence, as also evidenced by other authors, due to thrombogenic and hypoxic potential [7].

Finally, it is interesting to notice that on a multivariate analysis we have described a higher trend towards flap failure in maxillary reconstruction compared to mandibular ones. In univariate analysis, this trend reaches statistical significance (*p* < 0.001). Similarly, Brown et al. reported a higher rate of flap failure in upper jaw reconstruction [42]. We hypothesized that the longer distance from laterocervical vessels the higher the risk of pedicle kinking in maxillary reconstruction by means of a free flap. Furthermore, in these specific cases a vessel graft is more likely to be necessary, thus increasing the operative time. It is still argued whether the use of a vessel graft itself also increases the risk of endovascular thrombosis [43,44,45].

Some limitations of the present study must be highlighted. First, this analysis was clearly performed on a small number of patients. Nonetheless, the number of flap failure was quite consistent and allowed us to assess the possible role of risk factors. Secondly, the retrospective nature of this paper was a further weakness. Notwithstanding these limitations, the study suggests that smoking, reconstructive site (maxilla), the use of a vessel different from facial artery (especially due to oncological reasons) and previous surgical interventions might influence the success and survival of a free flap reconstruction. Factors affecting free flap survival and failure are important issues for future research and a prospective and multicentric assessment on a wider population is required.

## 5. Conclusions

Volume, length and number of fibula flap segments did not seem to influence the success of free flap reconstruction. To the best of our knowledge, the volume of the segments was investigated as a risk factor for the first time. On univariate analysis, the use of an artery different from the facial one as a recipient vessel and reconstruction site of the upper jaw seemed to impact on flap failure, while smoking habits and previous surgical interventions showed a higher trend.

## Figures and Tables

**Table 1:** Demographic, clinical and surgical data collected. ASA = American Society of Anesthesiologists; MRONJ = Medication-Related Osteo-Necrosis of the Jaws.

Variable		*n* = 71	%
Sex	Males	37	52.1
	Females	34	47.9
Smoke	Active smokers	15	21.1
	Former smokers	27	38
	Non-smokers	29	40.9
Alcohol consume	Yes	21	29.5
	No	50	70.5
Previous treatments	Previous surgery	33	46.5
	Previous chemotherapy	12	17.0
	Previous radiotherapy	16	22.5
ASA score	ASA I	9	12.7
	ASA II	37	52.1
	ASA III	24	33.8
	ASA IV	1	1.4
Diabetes mellitus	Yes	4	5.7
	No	67	94.3
Autoimmune diseases	Yes	5	7
	No	66	93
Indication for surgery	Malignant oncologic disease	32	45.1
	Osteoradionecrosis (ORN)	6	8.5
	Benign oncologic disease	17	23.9
	Others (MRONJ, atrophic jaws, vascular malformations)	16	22.5
Site of reconstruction	Mandible	47	66.2
	Maxilla	22	31.0
	Combined maxillo-mandibular reconstruction	2	2.8
Simultaneous neck dissection	Yes	18	25.4
	No	53	74.6
Simultaneous tracheotomy	Yes	47	66.2
	No	24	33.8
Recipient vein	Single anastomosis	26	36.6
	Double anastomosis	22	31.0
Recipient artery	Facial artery	49	69.0
	Other arteries (external carotid, superior thyroid artery, lingual artery, submandibular artery)	11	15.5

**Table 2:** Univariate analysis results. CI (LL) = Confidence Interval (Lower Limit); CI (UL) = Confidence Interval (Upper Limit); BMI = Body Mass Index; ASA = American Society of Anesthesiologists.

Parameter	Measured Variable	Odds Ratio	CI (LL)	CI (UL)	*p*-Value
Age	Continuous variable	1.010	0.975	1.047	0.575
Sex	Male:Female	2.148	0.650	7.094	0.210
Smoke	Non smokers vs. Active smoker	0.717	0.167	3.073	0.655
	Non-smoker vs. Ex-smoker	1.435	0.356	5.781	0.612
	Active smoker vs. Ex-smokers	2.000	0.419	9.551	0.385
BMI	Continuous variable	1.002	0.873	1.150	0.976
Hypertension	No vs. Yes	0.667	0.194	2.285	0.519
Diabetes mellitus	No vs. Yes	>999	<0.001	>999	0.979
Autoimmune diseases	No vs. Yes	0.368	0.056	2.434	0.210
ASA score	I vs. II	<0.001	<0.001	>999	0.895
	I vs. III	<0.001	<0.001	>999	0.887
	I vs. IV	1.000	<0.001	>999	1.000
	II vs. III	0.467	0.143	1.522	0.206
	II vs. IV	>999	<0.001	>999	0.965
	III vs. IV	>999	<0.001	>999	0.962
Preoperative radiotherapy	No vs. Yes	0.714	0.190	2.687	0.619
Previous surgery	No vs. Yes	0.706	0.225	2.213	0.550
Site	Mandible vs. Maxilla	0.089	0.024	0.333	<0.001 *
Neck dissection	No vs. Yes	0.650	0.188	2.52	0.497
Tracheotomy	No vs. Yes	0.545	0.152	1.955	0.352
Operation time	Continuous variable	1.002	0.998	1.006	0.425
Recipient artery	Facial artery vs. Other arteries	0.234	0.057	0.957	0.043 *
Venous anastomosis	Single vs. Double	2.815	0.644	12.306	0.169
Segment length	Continuous variable	0.680	0.329	1.405	0.297
Segment volume	Continuous variable	1.001	0.772	1.298	0.994
Number of used segments	1 segment vs. 2 segments	0.964	0.166	5.596	0.968
	1 segment vs. 3 segments	0.750	0.118	4.773	0.761
	1 segment vs. 4 segments	1.500	0.109	20.675	0.762
	2 segments vs. 3 segments	0.778	0.210	2.882	0.707
	2 segments vs. 4 segments	1.556	0.160	15.123	0.703
	3 segments vs. 4 segments	2.000	0.191	20.898	0.563

* Statistically significant.

## Data Availability

The data presented in this study are available on request from the corresponding author. The data are not publicly available due to privacy restrictions.
